# The role of mathematical modelling in malaria elimination and eradication (Comment on: Can malaria be eliminated?)

**DOI:** 10.1016/j.trstmh.2009.01.027

**Published:** 2009-06

**Authors:** Richard J. Maude, Wirichada Pontavornpinyo, Sompob Saralamba, Arjen M. Dondorp, Nicholas P.J. Day, Nicholas J. White, Lisa J. White

**Affiliations:** a Centre for Clinical Vaccinology and Tropical Medicine, Nuffield Department of Clinical Medicine, John Radcliffe Hospital, University of Oxford, Oxford, UK; b Faculty of Tropical Medicine, Mahidol University, Bangkok, Thailand

For many countries, as Professor Greenwood points out, the answer to his question is a resounding ‘yes’.^1^ Perhaps the more pertinent question is, with so many more interventions now available, ‘how?’. The two most important new tools that have been added to our malaria control arsenal since the 1950s are artemisinin combination therapies and insecticide-treated bed nets. These are most likely to be effective when used together, in combination with indoor residual spraying where there are suitable vectors. The problem we are faced with is a lack of experience of using such an elimination strategy on a large scale, leading to a lack of data, particularly on the best way to roll out ACTs. In the past, mass screening and treatment, mass drug administration and large-scale replacement of first-line therapies have all been attempted, with varying degrees of success. In the context of newly arisen artemisinin resistance and the diminishing effectiveness of the pyrethroid insecticides, particular care must be taken to preserve the effectiveness of these compounds for as long as possible, whilst achieving maximum impact in a wide variety of epidemiological settings.

Another powerful tool that we did not have in the 1950s is sophisticated computer-based mathematical modelling. This facilitates the use of the limited data currently available to make predictions about future events. In other words, it provides a rational framework on which to base decisions made using limited but diverse and complex inter-related information, such as population demographics, pharmacokinetics and pharmacodynamics, treatment-seeking behaviour, spatially distributed risk factors, the presence of antimalarial resistance, etc. Identifying, preparing and calibrating such data sets for each country for the application of mathematical models is a huge challenge in itself.^2^ The accuracy of modelling predictions improves in an iterative process as more data become available. Hence, the more intense the malaria elimination efforts are and as long as data are rigorously collected, the more useful and informative modelling becomes. The Bill and Melinda Gates Foundation and others have realized the potential of mathematical modelling to help guide malaria elimination and eradication. Malaria elimination/eradication modelling is still in its infancy (a PubMed search for mathematical modelling papers with the keywords ‘malaria’, ‘elimination’ or ‘eradication’ produced only two articles)^3^, ^4^ so these groups have provided significant funding to focus modelling efforts on this problem.

Professor Greenwood states that new tools are likely to be required in order to eliminate malaria in high-transmission areas.^1^ If, eventually, we do have the luxury of an effective malaria vaccine or powerful new gametocytocidal drugs they are likely to be expensive and their scale-up will inevitably be based on limited trial data in the initial stages. Mathematical modelling will be indispensible in helping to maximize their impact.

## Funding

Mahidol-Oxford Tropical Medicine Research Unit is funded by the Wellcome Trust of Great Britain.

## Conflicts of interest

None declared.

## Ethical approval

Not required.
